# Maize Yield Response to Water Supply and Fertilizer Input in a Semi-Arid Environment of Northeast China

**DOI:** 10.1371/journal.pone.0086099

**Published:** 2014-01-20

**Authors:** Guanghua Yin, Jian Gu, Fasheng Zhang, Liang Hao, Peifei Cong, Zuoxin Liu

**Affiliations:** 1 Institute of Applied Ecology, Chinese Academy of Sciences, Shenyang, China; 2 University of Chinese Academy of Sciences, Beijing, China; Cairo University, Egypt

## Abstract

Maize grain yield varies highly with water availability as well as with fertilization and relevant agricultural management practices. With a 311-A optimized saturation design, field experiments were conducted between 2006 and 2009 to examine the yield response of spring maize (Zhengdan 958, *Zea mays* L) to irrigation (*I*), nitrogen fertilization (total nitrogen, urea-46% nitrogen,) and phosphorus fertilization (P_2_O_5_, calcium superphosphate-13% P_2_O_5_) in a semi-arid area environment of Northeast China. According to our estimated yield function, the results showed that *N* is the dominant factor in determining maize grain yield followed by *I*, while *P* plays a relatively minor role. The strength of interaction effects among *I*, *N* and *P* on maize grain yield follows the sequence *N*+*I* >*P*+*I*>*N*+*P*. Individually, the interaction effects of *N*+*I* and *N*+*P* on maize grain yield are positive, whereas that of *P*+*I* is negative. To achieve maximum grain yield (10506.0 kg·ha^−1^) for spring maize in the study area, the optimum application rates of *I*, *N* and *P* are 930.4 m^3^·ha^−1^, 304.9 kg·ha^−1^ and 133.2 kg·ha^−1^ respectively that leads to a possible economic profit (*EP*) of 10548.4 CNY·ha^−1^ (CNY, Chinese Yuan). Alternately, to obtain the best *EP* (10827.3 CNY·ha^−1^), the optimum application rates of *I*, *N* and *P* are 682.4 m^3^·ha^−1^, 241.0 kg·ha^−1^ and 111.7 kg·ha^−1^ respectively that produces a potential grain yield of 10289.5 kg·ha^−1^.

## Introduction

Maize is the third most important grain crop after rice and wheat grown in China [1], [2]. In order to ensure food security for its vast population, the Chinese government and its research institutions have made extensive efforts to improve maize grain production in North China since the 1950s [3−5].

Water scarcity and soil infertility are two critical factors limiting maize grain yield over most regions of North China [6−8]. Although irrigation and fertilization are widely applied to improve maize productivity [9], [10], maize production in China has not been able to keep pace with grain demand [11], [12]. At the same time, low water use efficiency aggravates water stress in North China [13−15] while excessive inputs of chemical fertilizer result in surplus nitrogen and phosphorus in soils that cause eutrophication of surface water as well as greenhouse gas emissions [16−20]. In modern agriculture, such consequences arise mainly from a limited understanding of how irrigation and fertilization affect maize production and a biased estimation of the yield function for identifying maize yield variation. In this context, there is a need to investigate the combined effect of water supply and fertilizer input on maize productivity in North China.

Many field studies have been conducted since the 1990s to examine main and interaction effects of irrigation and fertilization on maize productivity around the world, including North China [21−26]. The optimum coupling or combination of water supply and fertilizer inputs has been derived to seek maximum maize grain yield or to achieve maximum water and fertilizer use efficiency [27−30]. However, these studies mostly focused on the individual influences of irrigation (*I*), nitrogen application (*N*), phosphorus application (*P*) and/or their binary combination effects on maize productivity. A holistic understanding of the ternary combination effect of *I*, *N* and *P* on maize productivity is still developing. The economic efficiency of growing maize is another important factor influencing maize grain production [31−34]. Farmers will grow more maize if the economic profits of growing maize are higher than for other crops. Profits associated with maize production, however, decrease with improper management practices as well as with increasing energy, material and human labor costs in the context of global climate change [35−39]. The declining profit rate dampens farmers’ enthusiasm for growing maize and consequently impacts maize grain production [40], [41]. Thus, it is important to improve maize productivity while taking into account the economic evaluation of growing maize.

The relationship between maize grain yield and management practices varies over time and space depending on the maize cultivars, climatic conditions and cropping systems. Knowledge obtained from studies in other regions may not be valid in any specific area of North China. Therefore, the objectives of this study were (1) to construct a yield function to examine the combination effect of *I*, *N* and *P* on maize productivity using field experimental data collected from 2006 to 2009 in a semi-arid environment of Northeast China and (2) to use the estimated yield function for further deriving optimum application rates of *I*, *N* and *P* based on the criteria of maximum grain yield and best economic profit.

## Materials and Methods

### Site and Soil

**Figure 1:**
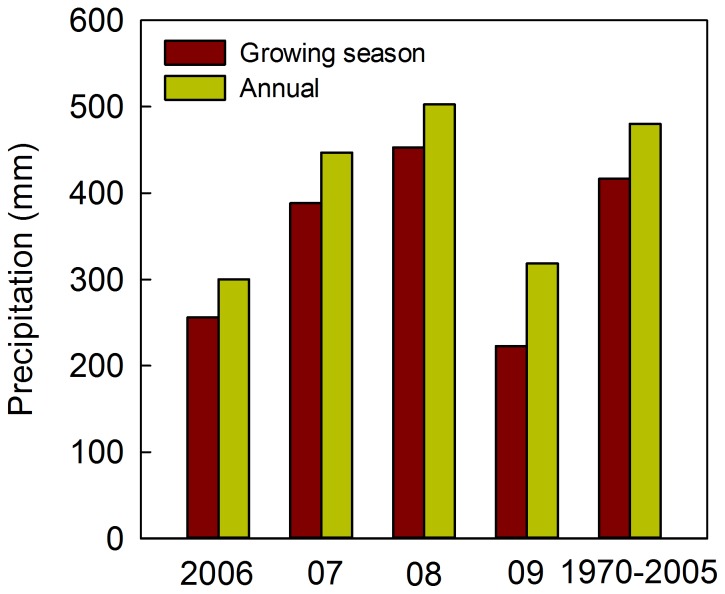
Annual precipitation and precipitation during maize growing season during 2006–2009 in Fuxin County.

The field study was conducted from 2006 to 2009 at the field experimental station of Liaoning Key Laboratory of Water-Saving Agriculture in Fuxin County of Northeast China (42°08′14″ N, 121°44′21″ E). This region is a warm temperate zone with a temperate continental monsoon climate. According to the Fuxin Weather Station, the average annual temperature is 7.2°C with an average of 2865.5 hrs of annual sunshine. It is a typical semi-arid area with average annual precipitation of 480 mm, over 60% of which occurs from June to August. The compensation of water resources depends mainly on precipitation of atmosphere. Annual precipitation and precipitation during the maize growing season of Fuxin County are shown in Figure 1.

The main agricultural soil in the region is cinnamon soil which develops through a combination of calcium carbonate leaching, illuviation and humification. It is characterized by a thin humus layer and a medium or thick solum. Its bulk density is 1.51 g·cm^−3^, pH (H_2_O) is 7.5−8.5 and the average soil organic matter content is 10.2 g·kg^−1^. The average soil total nitrogen and available phosphorus concentrations are 6.1 g·kg^−1^ and 4.0 g·kg^−1^, respectively.

### Experimental Design and Treatments

To reduce cost and size, the experiment in this study was implemented according to a 311-A optimized saturation design [42]. This system consisted of three factors at five levels. There were 11 treatments with 3 replicates each for a total of 33 experimental plots. Each experimental plot was 40 m^2^ in size (10 m×4 m). The water supply and fertilizer inputs were standardized for comparability by applying a non-dimensional linear code substitution (Table 1). Rates of *N* and *P* in Table 1 were expressed in format of pure nitrogen and P_2_O_5_ that were supplemented by urea (46% total nitrogen) and calcium superphosphate (13% P_2_O_5_). One unit of *I*, *N* and *P* represented 225 m^3^·ha^−1^ of *I*, 112.5 kg·ha^−1^ of total nitrogen and 67.5 kg·ha^−1^ of P_2_O_5_, which indicated 225 m^3^·ha^−1^ of water supply, 244.6 kg·ha^−1^ of urea and 519.2 kg·ha^−1^ of calcium superphosphate, respectively. One third of the urea used in each treatment was applied at the sowing stage and the remaining amount at the early jointing stage. All calcium superphosphate was applied at the sowing stage in each treatment. Experimental plots were variously irrigated at the jointing stage. All necessary permits were obtained for the described field experiments. The land user and owner approved the field-work activities at each experiment plot. The field employed in this study is not protected in any way, and the study did not involve any endangered or protected species.

**Table 1:** Experimental treatments of maize using a 311-A optimized saturation design during 2006–2009 in Fuxin County.

Treatments	Code level			Application rate		
	*I* †	*N*	*P*	*I*	*N*	*P*
	*X*_1_ ‡	*X*_2_	*X*_3_	m^3^·ha^−1^	kg·ha^−1^	kg·ha^−1^
1	2	0	0	900	225	135
2	−2	0	0	0	225	135
3	1	−1.414	−1.414	675	66	39.54
4	1	1.414	−1.414	675	384	39.54
5	1	−1.414	1.414	675	66	230.46
6	1	1.414	1.414	675	384	230.46
7	−1	2	0	225	450	135
8	−1	−2	0	225	0	135
9	−1	0	2	225	225	270
10	−1	0	−2	225	225	0
11	0	0	0	450	225	135

*I*, *N* and *P* are abbreviations for irrigation, nitrogen fertilization and phosphorus fertilization, respectively.

*X*_1_, *X*_2_ and *X*_3_ are non-dimensional linear code for irrigation, nitrogen fertilization and phosphorus fertilization, respectively.

### Data Collection and Analysis

The maize cultivar in this experiment was Zhengdan 958 (*Zea mays* L). In China, the planting area of Zhengdan 958 was 4.54 million ha in 2009, and the planting area of this variety is still the largest in 2012 [43]. Zhengdan 958 has outstanding yield performance. It can generate relatively stable yield under various environmental conditions and has good disease resistance. This variety can be planted in high density and the ideal planting density is 60,000 to 75,000 plants per ha. In this study, maize plant density was 60,000 plants per ha with 50 cm between rows. The maize was planted in late April and harvested in late September. At maize maturity, the outer two rows in each experimental plot were considered as edge effects and not harvested, while the remaining middle rows were hand-harvested for analysis of maize grain yield. The effective area of each experimental plot was approximately 20 m^2^. The average fresh ear weight (*G*_1_, kg) of each treatment was estimated and the average grain yield, Y (kg·ha^−1^), was computed as:
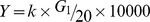
where *k* is the ratio of grain dry weight to fresh ear weight for each treatment. To estimate the values of *k*, ten medium-sized ears were sampled from each experimental plot and their average fresh ear weight (*G*_2_, kg) and average fresh grain weight (*G*_3_, kg) were measured for each treatment The grain of Zhengdan 958 dries slowly before harvest. Extra moisture in maize grain should be removed before estimating the average maize grain yield. The average moisture content of fresh grain for each treatment (*A*%) was therefore determined using a PM-8188 Grain Moisture Tester (Japan). Then *k* was calculated as:



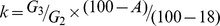



A quadratic regression orthogonal design was used to construct the yield function in this study. Regression analysis was conducted by R software and the results were presented in graphs by SigmaPlot 10.0.

## Results and Discussion

### Yield Function

The average maize grain yield of each treatment from 2006 to 2009 varied between 8468.2 kg·ha^−1^ and 10478.2 kg·ha^−1^. The best-fitted yield function, which quantified the relationship between maize grain yield (Y*_INP_*) and the code levels of *I*, *N* and *P* (i.e., *X_1_*, *X_2_*, *X_3_*), was expressed as:
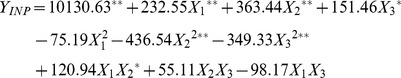
(1)where the coefficient of determination (*R^2^*) for the regression is 0.87 at the significance level of *P*<0.01 (*F*-test). The reliability of coefficients for *X_1_*, *X_2_* and *X_3_* are tested by a *t*-test. The symbol “**” stands for the significance at the level of *P*<0.01 and the symbol “*” for the significance at the level of *P*<0.05. In addition, the coefficients of *X*_1_^2^, *X*_2_ *X*_3_ and *X*_1_ *X*_3_ were significant at *P*<0.20, *P*<0.40 and *P*<0.10. Considering the importance of *I*, *N* and *P* on maize growth, the parameters in equation (1) were all taken into account when estimating maize grain yield in this study.

The code levels of *I*, *N* and *P* were proportional to their actual rates in each treatment. Thus, equation (1) appropriately described the combination effects of water spply and fertilizer input on maize grain yield in the semi-arid area examined in this study. It accounted for 87% of the variation in maize grain yield. Because of its accuracy and explicitness, equation (1) was used to analyze the main and individual effects as well as the interaction effects of *I*, *N* and *P* on maize grain yield (Text S1).

The main effects of *I*, *N* and *P* on maize grain yield were evaluated by comparing their corresponding coefficients in equation (1). The positive coefficients for *X*_1_, *X*_2_ and *X*_3_ suggested that *I*, *N* and *P* all had positive effects on maize grain yield. The largest value was observed for the coefficient of *X*_2_, indicating that *N* was the dominant factor influencing maximum maize grain yield. Similarly, *I* was recognized as a secondary factor determining maize grain yield, whereas *P* was a relatively minor factor. These results were partially consistent with findings from other studies around the world. Nitrogen input has a large effect on maize grain yield because maize production is an extractive process, with removal of maize equating to removal of nitrogen from the soil [44], [45]. The significant effect of water supply on maize grain yield in arid and semi-arid regions is ubiquitous and easily understood. It should be noted that the removal of phosphorus from agricultural soils by maize harvesting was also apparent in this study. It is well-established that supplemental phosphorus can significantly improve maize grain yield as well [7], [9], [17], [22]. In our study, the decreased response of maize grain yield to *P* compared to *N* and *I* may be due to the dry soil conditions which are characteristics of semi-arid areas in Northeast China.

To examine the individual effects of *I*, *N* and *P* on maize grain yield, each factor was selected as an independent variable with the other two factors fixed at 0 in equation (1) (Text S1). Then a subset of equations of yield function was derived respectively as:
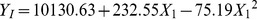
(2)

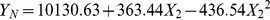
(3)

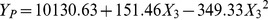
(4)


**Figure 2:**
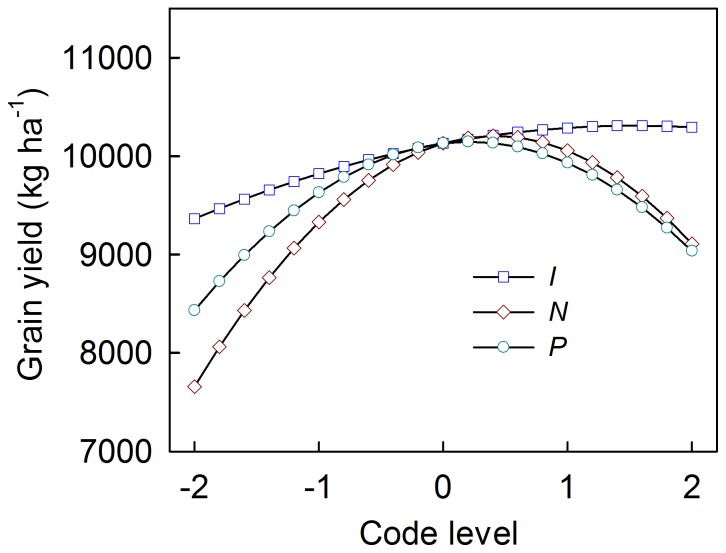
The individual effect of *I*, *N* and *P* on maize grain yield by fixing two factors at 0 level. *I*, *N* and *P* represent irrigation, nitrogen application and phosphorus application, respectively.

The individual effects of *I*, *N* and *P* on maize grain yield derived from equations (2) ∼ (4) are presented in Figure 2. The relationships between maize grain yield and *I*, *N* and *P* can be modeled using second-order parabolic equations. Apexes were observed when examining the trend of maize grain yield as the rates of *I*, *N* and *P* increased. Before the apex, maize grain yield increased as the rates of *I*, *N* and *P* increased. After the apex, maize grain yield decreased as the rates of *I*, *N* and *P* increased. These findings indicate that there must be optimum application rates of *I*, *N* and *P* when implementing agricultural management practices to improve maize grain yield.

**Figure 3:**
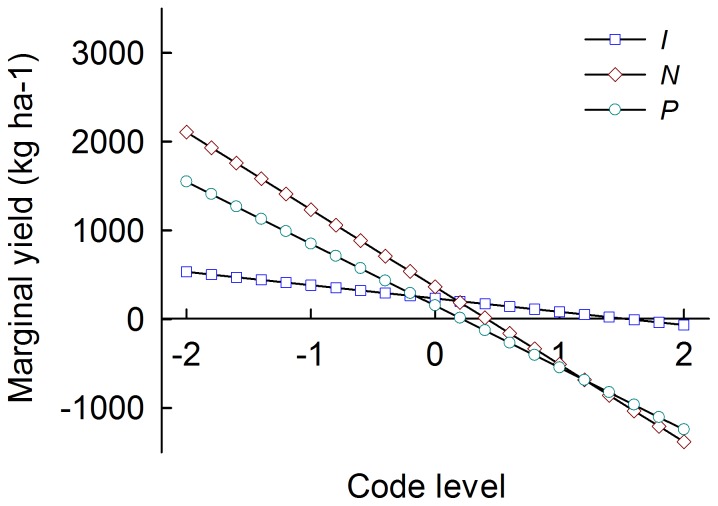
Marginal grain yield with respect to *I*, *N* and *P* through the first-order differential analysis of equations (2)∼(4). *I*, *N* and *P* represent irrigation, nitrogen application and phosphorus application, respectively.

The optimum application rates of *I*, *N* and *P* as individual influencing factors on maize grain yield were determined using marginal yield curves (Figure 3), which were the first-order differential analysis of equations (2) ∼ (4). The marginal yield showed a monotonic descending trend with the increasing rates of *I*, *N* and *P* and had intersections with the x-axis. The intersection points revealed the optimum application rates of *I*, *N* and *P*. The code values at the intersecting points were +1.546, +0.416 and +0.217 for *I*, *N* and *P*, respectively. According to Table 1, they represented 797.9 m^3^·ha^−1^ of *I*, 271.8 kg·ha^−1^ of *N* and 149.6 kg·ha^−1^ of *P*. In addition, levels of the majority of marginal yields before the intersections with the x-axis followed the sequence *N* >*P*> *I*. This suggests that the maize grain yield increased most with an increasing application rate of *N* compared to the increases of *I* and *P*. Maize grain yield was more sensitive to *P* than *I*.

**Figure 4:**
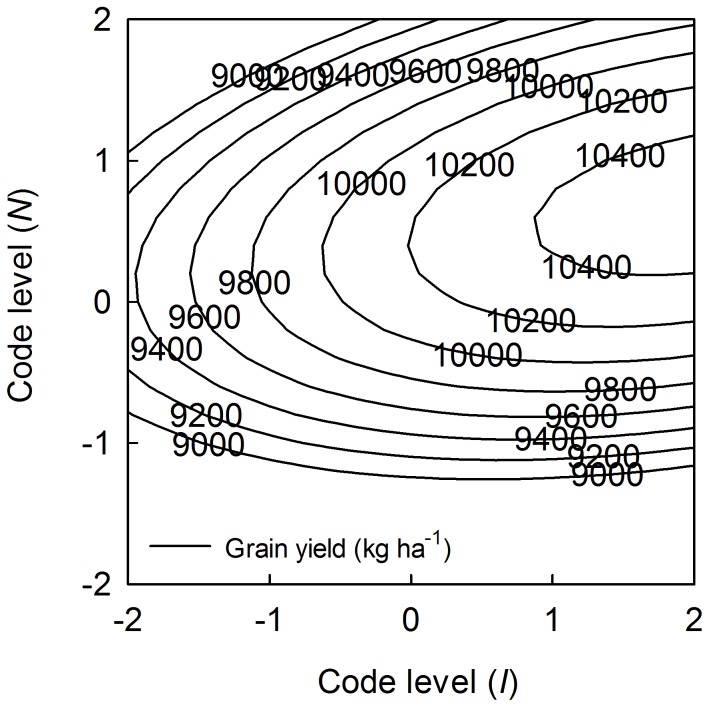
Interaction effects of *I* and *N* on maize grain yield by fixing *P* at 0 level. *I* and *N* represent irrigation and nitrogen application, respectively.

**Figure 5:**
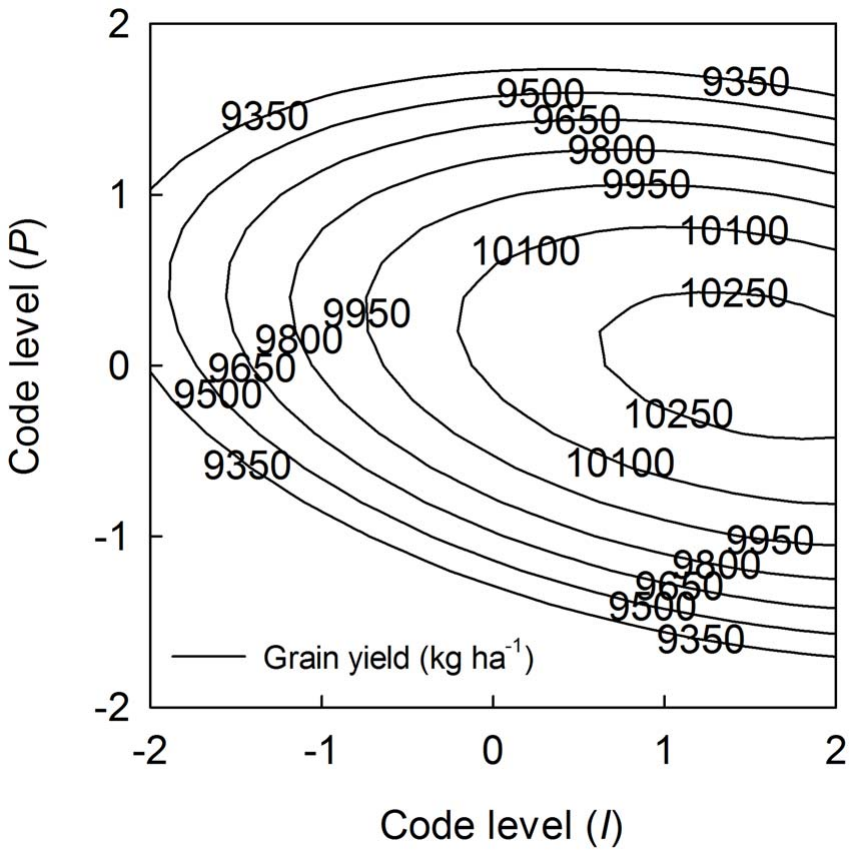
Interaction effects of *I* and *P* on maize grain yield by fixing *N* at 0 level. *I* and *P* represent irrigation and phosphorus application, respectively.

The interaction effects of *I*, *N* and *P* on maize grain yield were all less than their main effects in equation (1). By comparing the absolute values of the coefficients of *X*_1_ *X*_2_, *X*_1_ *X*_3_ and *X*_2_ *X*_3_, the strength of interaction effects of *I*, *N* and *P* on maize grain yield followed the sequence *N*+*I* >*P*+*I*>*N*+*P*. The coefficient for *X*_1_ *X*_3_ was positive, indicating that the interaction of *I* and *N* had a positive effect on maize grain yield. This finding is presented in Figure 4, which was constructed by fixing *P* at 0 level. In general, the maize grain yield increased as the application rates of *I* and *N* increased: an increase of any one input stimulates maize growth and creates a need for the other input. The optimum application rates of *I* and *N* for the highest grain yield (10,505.7 kg·ha^−1^) were at code levels of +2.117 and +0.710, which corresponded to 926.3 m^3^·ha^−1^ of *I* and 304.9 kg·ha^−1^ of *N*. The negative coefficient for *X*_1_ *X*_3_ suggested that the interaction of *I* and *P* was antagonistic and had an inhibitory effect on maize grain yield. This relationship is presented in Figure 5, which was constructed by fixing *N* at 0 level. At code levels of +1.547 and −0.001, equal to 798.1 m^3^·ha^−1^ of *I* and 134.9 kg·ha^−1^ of *P*, respectively, maize grain yield reached its highest value of 10,310.4 kg·ha^−1^. Although the coefficient of *X*_2_ *X*_3_ was positive, the interaction of *N* and *P* on maize grain yield was similar to that of *I* and *N*, but weaker and not significant at a relatively high level. This partly reveals the importance of water supply in maize growth in semi-arid areas. No contour plot of this interaction is presented here, although the interaction between *N* and *P* on maize grain yield was used to estimate the optimum schemes in next part.

### Optimum Schemes

The findings presented above indicate the complex and sometimes antagonistic interactions of water supply and fertilizer input on maize productivity in semi-arid areas of Northeast China. The maize grain yield does not always increase as the additions of *I*, *N* and *P* improve. The optimum application rates of *I*, *N* and *P* were estimated to obtain the maximum grain yield and best economic profit.

To maximize maize grain yield, we set the first-order partial derivatives of equation (1) to zero (Text S1):
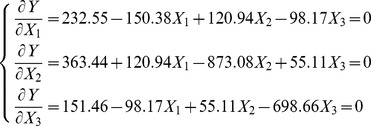



By solving the above equations, the maximum maize grain yield, 10506.0 kg·ha^−1^, can be obtained when the code levels of *I*, *N* and *P* were at +2.135, +0.710 and −0.027, respectively. According to Table 1, the optimum rates of *I*, *N* and *P* for the maximum maize grain yield were 930.4 m^3^·ha^−1^, 304.9 kg·ha^−1^ and 133.2 kg·ha^−1^, respectively.

Compared to the optimum application rates of *I*, *N* and *P* based on single-factor and binary combination effects, the optimum application rates based on the ternary combination effect have higher demand for *I* and *N* and less demand for *P*. This should be attributed to the effects of complex interactions of *I*, *N* and *P* on maize grain yield. In addition, maize grain yield showed different sensitivities to *I*, *N* and *P* in Figure 3. In the case of the ternary combination, these factors were considered holistically when estimating the maximum of the yield function. The optimum scheme indicated by the ternary combination enabled farmers to maximize maize grain yield by adjusting management practices without considering the cost of maize growing.

The increase of water supply and fertilizer input may increase maize grain yield as well as the economic profit. The economic profit of growing maize, *EP,* is decided by the relationship between outputs (i.e., the maize grain yield times the price of maize grain) and inputs (i.e., cost of irrigation, fertilization and seed). This relationship can be expressed as:
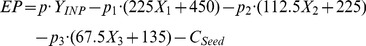
(5)where *p* stands for the price of maize grain (1.3 CNY⋅kg^−1^, CNY, Chinese Yuan), and *p*_1_, *p*_2_ and *p*_3_ for the prices of water (0.750 CNY⋅m^−3^, i.e., electricity and labor fees for irrigation), urea (3.913 CNY⋅kg^−1^) and calcium superphosphate (5.769 CNY⋅kg^−1^), respectively. Note that the prices of urea and calcium superphosphate per kilogram were calculated from the cost of them to provide a kilogram of pure total nitrogen and P_2_O_5_. *C_Seed_* represents the cost of maize seeds for each treatment in this study (450 CNY⋅ha^−1^). To obtain the maximum economic profit, the highest value of equation (5) was solved using the method of first-order partial derivatives as well. The results show that the highest economic profit (10827.3 CNY⋅ha^−1^) was obtained when *I*, *N* and *P* were at code levels of 1.033, 0.142 and −0.345, respectively. This optimum scheme consisted of a ternary combination of *I*, *N* and *P* at rates of 682.4 m^3^⋅ha^−1^, 241.0 kg⋅ha^−1^ and 111.7 kg⋅ha^−1^, respectively.

To further discern the results, we compared the two optimum schemes based on the maximum grain yield and the best economic profit. The economic profit of the optimum scheme for maximum grain yield (10506.0 kg·ha^−1^) was 10548.4 CNY⋅ha^−1^ while the grain yield of the optimum scheme for the best economic profit (10827.3 CNY⋅ha^−1^) was 10289.5 kg·ha^−1^. Notwithstanding a little higher yield, the optimum scheme for maximum grain yield produced less economic profit than that of the optimum scheme for the best economic profit. In addition, the optimum scheme for maximum grain yield consumed more water and fertilizer than the optimum scheme for the best economic profit. This apparently will increase demand on water and mineral resources and result in leaching of surplus nitrogen and phosphorus into soil and water environments. In contrast, the optimum scheme for the best economic profit produced both relatively high grain yield and economic profit while consumed relatively less water and fertilizer. Considering the water resource and soil conditions, the optimum scheme for the best economic profit was therefore more acceptable and should be recommended in the study area.

## Conclusions

Yield response of spring maize (Zhengdan 958, *Zea mays* L) to water supply and fertilizer input in a semi-arid area of Northeast China was studied. In this field experiment, *I*, *N* and P as well as their interaction effects had significant influences on maize grain yield. The yield function derived by the ternary combination was able to describe these influences holistically. To obtain maximum maize grain yield (10506.0 kg·ha^−1^) in the semi-arid areas examined in this study, the optimum application rates of *I*, *N* and *P* based on the present findings were 930.4 m^3^·ha^−1^, 304.9 kg·ha^−1^ and 133.2 kg·ha^−1^, respectively. Alternately, to obtain the best economic profit (10827.3 CNY⋅ha^−1^), the optimum application rates of *I*, *N* and *P* were 682.4 m^3^·ha^−1^, 241.0 kg·ha^−1^ and 111.7 kg·ha^−1^, respectively. The latter scheme is recommended in the study area because of its relatively high grain yield and economic profit performance.

## Supporting Information

Text S1 **A brief interpretation of formulas employed in the manuscript.**
(DOCX)

## References

[1] Li J (2009) Production, breeding and process of maize in China. In: Bennetzen JL, Hake SC, editors. Handbook of maize: Its biology. New York: Springer. pp. 563–576.

[2] Ray DK, Mueller ND, West PC, Foley JA (2013) Yield trends are insufficient to double global crop production by 2050. PLoS ONE 8: e66428. 23840465 10.1371/journal.pone.0066428 PMC3686737

[3] Wang H, Zhang M, Cai Y (2009) Problems, challenges, and strategic options of grain security in China. Advances in Agronomy 103: 101–147.

[4] Wang T, Ma X, Li Y, Bai D, Liu C, et al (2011) Changes in yield and yield components of single-cross maize hybrids released in China between 1964 and 2001. Crop Science 51: 512–525.

[5] Ci X, Li M, Xu J, Lu Z, Bai P, et al (2012) Trends of grain yield and plant traits in Chinese maize cultivars from the 1950s to the 2000s. Euphytica 183: 1–12.

[6] Zou C, Gao X, Shi R, Fan X, Zhang F (2008) Micronutrient deficiencies in crop production in China. In: Alloway BJ, editor. Micronutrient deficiencies in global crop production. The Netherlands: Springer. pp. 127–148.

[7] Wang CT, Li SK (2010) Assessment of limiting factors and techniques prioritization for maize production in China. Scientia Agricultura Sinica 43: 1136–1146 (in Chinese)..

[8] Zhang F, Yin G, Wang Z, McLaughlin N, Geng X, et al (2013) Quantifying spatial variability of selected soil trace elements and their scaling relationships using multifractal techniques. PLoS ONE 8: e69326.9. 23874944 10.1371/journal.pone.0069326 PMC3706377

[9] Fan T, Stewart BA, Wang Y, Luo J, Zhou G (2005) Long–term fertilization effects on grain yield, water–use efficiency and soil fertility in the dryland of Loess Plateau in China. Agriculture, Ecosystems & Environment 106: 313–329.

[10] Khan S, Hanjra MA, Mu J (2009) Water management and crop production for food security in China: A review. Agricultural Water Management 96: 349–360.

[11] Neumann K, Verburg PH, Stehfest E, Müller C (2010) The yield gap of global grain production: A spatial analysis. Agricultural Systems 103: 316–326.

[12] Meng Q, Hou P, Wu L, Chen X, Cui Z, et al (2013) Understanding production potentials and yield gaps in intensive maize production in China. Field Crops Research 143: 91–97.

[13] Liu Z, Gao P (2009) Application basis and technology of water-saving agriculture in semi-arid areas of Northeast China. Beijing: Science Press. p. 314.

[14] Fang Q, Ma L, Yu Q, Ahuja LR, Malone RW, et al (2010) Irrigation strategies to improve the water use efficiency of wheat–maize double cropping systems in North China Plain. Agricultural Water Management 97: 1165–1174.

[15] Bernacchi CJ, Hollinger SE, Meyers T (2005) The conversion of the corn/soybean ecosystem to no-till agriculture may result in a carbon sink. Global Change Biology 11: 1867–1872.

[16] Wang X, Willms WD, Hao X, Zhao M, Han G (2010) Cultivation and reseeding effects on soil organic matter in the mixed prairie. Soil Science Society of America Journal 74: 1348–1355.

[17] Ma W, Ma L, Li J, Wang F, Sisák I, et al (2011) Phosphorus flows and use efficiencies in production and consumption of wheat, rice, and maize in China. Chemosphere 84: 814–821. 21570104 10.1016/j.chemosphere.2011.04.055

[18] Wang X, Wang J, Zhang J (2012) Comparisons of three methods for organic and inorganic carbon in calcareous soils of northwestern China. PLoS ONE 7: e44334. 22952957 10.1371/journal.pone.0044334 PMC3432125

[19] Wang X, Gan YT, Hamel C, Lemke RL, McDonald CL (2012) Water use profiles across the rooting zones of various pulse crops. Field Crops Research 134: 130–137.

[20] Cui Z, Yue S, Wang G, Meng Q, Wu L, et al (2013) Closing the yield gap could reduce projected greenhouse gas emissions: a case study of maize production in China. Global Change Biology 19: 2467–2477. 23553871 10.1111/gcb.12213

[21] Jokela WE (1992) Nitrogen fertilizer and dairy manure effects on corn yield and soil nitrate. Soil Science Society of America Journal 56: 148–154.

[22] Wortmann CS, Dobermann AR, Ferguson RB, Hergert GW, Shapiro CA, et al (2009) High–yielding corn response to applied phosphorus, potassium, and sulfur in Nebraska. Agronomy Journal 101: 546–555.

[23] Ju X, Christie P (2011) Calculation of theoretical nitrogen rate for simple nitrogen recommendations in intensive cropping systems: A case study on the North China Plain. Field Crops Research 124: 450–458.

[24] Hou P, Gao Q, Xie R, Li S, Meng Q, et al (2012) Grain yields in relation to N requirement: Optimizing nitrogen management for spring maize grown in China. Field Crops Research 129: 1–6.

[25] Jin L, Cui H, Li B, Zhang J, Dong S, et al (2012) Effects of integrated agronomic management practices on yield and nitrogen efficiency of summer maize in North China. Field Crops Research 134: 30–35.

[26] Hu H, Ning T, Li Z, Han H, Zhang Z, et al (2013) Coupling effects of urea types and subsoiling on nitrogen–water use and yield of different varieties of maize in northern China. Field Crops Research 142: 85–94.

[27] Dang TH, Cai GX, Guo SL, Hao MD, Heng LK (2006) Effect of nitrogen management on yield and water use efficiency of rainfed wheat and maize in Northwest China. Pedosphere 16: 495–504.

[28] Liu WZ, Zhang XC (2007) Optimizing water and fertilizer input using an elasticity index: a case study with maize in the loess plateau of china. Field Crops Research 100: 302–310.

[29] El-Hendawy SE, Schmidhalter U (2010) Optimal coupling combinations between irrigation frequency and rate for drip–irrigated maize grown on sandy soil. Agricultural Water Management 97: 439–448.

[30] Wang X, Dai K, Zhang D, Zhang X, Wang Y, et al (2011) Dryland maize yields and water use efficiency in response to tillage/crop stubble and nutrient management practices in China. Field Crops Research 120: 47–57.

[31] Grassini P, Cassman KG (2012) High–yield maize with large net energy yield and small global warming intensity. Proceedings of the National Academy of Sciences 109: 1074–1079. 10.1073/pnas.1116364109 PMC3268318 22232684

[32] Mulwa R, Emrouznejad A, Muhammad L (2009) Economic efficiency of smallholder maize producers in Western Kenya: a DEA meta–frontier analysis. International Journal of Operational Research 4: 250–267.

[33] Rahman S, Rahman MS, Rahman MH (2012) Joint determination of the choice of growing season and economic efficiency of maize in Bangladesh. Journal of the Asia Pacific Economy 17: 138–150.

[34] Sharma AR, Singh R, Dhyani SK, Dube RK (2011) Agronomic and economic evaluation of mulching in rainfed maize–wheat cropping system in the Western Himalayan region of India. Journal of Crop Improvement 25: 392–408.

[35] Guo R, Lin Z, Mo X, Yang C (2010) Responses of crop yield and water use efficiency to climate change in the North China Plain. Agricultural Water Management 97: 1185–1194.

[36] Xiong W, Holman I, Lin E, Conway D, Jiang J, et al (2010) Climate change, water availability and future cereal production in China. Agriculture, Ecosystems & Environment 135: 58–69.

[37] Piao S, Ciais P, Huang Y, Shen Z, Peng S, et al (2010) The impacts of climate change on water resources and agriculture in China. Nature 467: 43–51. 20811450 10.1038/nature09364

[38] Li X, Takahashi T, Suzuki N, Kaiser HM (2011) The impact of climate change on maize yields in the United States and China. Agricultural Systems 104: 348–353.

[39] Tao F, Zhang Z (2011) Impacts of climate change as a function of global mean temperature: maize productivity and water use in China. Climatic Change 105: 409–432.

[40] Tong C, Hall CAS, Wang H (2003) Land use change in rice, wheat and maize production in China (1961–1998). Agriculture, Ecosystems & Environment 95: 523–536.

[41] Owombo PT, Akinola AA, Ayodele OO, Koledoye GF (2012) Economic impact of agricultural mechanization adoption: Evidence from maize farmers in Ondo State, Nigeria. Journal of Agriculture and Biodiversity Research 1: 25–32.

[42] Ding XQ (1986) The applied regression design of the field experiment. Jilin Science&Technology Press. 199–203. (in Chinese).

[43] Jin X, Fu Z, Ding D, Li W, Liu Z, et al (2013) Proteomic identification of genes associated with maize grain-filling rate. PLoS ONE 8: e59353. 23527170 10.1371/journal.pone.0059353 PMC3601958

[44] Lawlor DW, Lemaire G, Gastal F (2001) Nitrogen, plant growth and crop yield. In: Lea PJ, Morot-Gaudry JF, editors. Plant nitrogen. Berlin Heidelberg: Springer. pp. 343–367.

[45] Peng Y, Li X, Li C (2012) Temporal and spatial profiling of root growth revealed novel response of maize roots under various nitrogen supplies in the field. PLoS ONE 7: e37726. 22624062 10.1371/journal.pone.0037726 PMC3356300

