# Supplementary material for: Maize Yield Response to Water Supply and Fertilizer Input in a Semi-Arid Environment of Northeast China
Source: PLoS One. 2014 Jan 20;9(1):e86099. doi: 10.1371/journal.pone.0086099 (PMC3896526; doi:10.1371/journal.pone.0086099)
Supplement: Text S1 — A brief interpretation of formulas employed in the manuscript. (DOCX) [file pone.0086099.s001.docx]

**Text S1:** **A brief interpretation of the formulas employed in the manuscript**

**Equation (1): Main effect**

Equation (1) described how *X*_1_, *X*_2_ and *X*_3_ (i.e., *I*, *N* and *P*) influence maize grain yield of experimental plots during 2006-2009 in Fuxin County. The coefficients of *X*_1_, *X*_2_ and *X*_3_ indicate the main effects of *I*, *N* and *P* in determining maize grain yield. The coefficients of *X*_1_*X*_2_, *X*_2_*X*_3_ and *X*_1_*X*_3_ suggest the interaction effects of *I*, *N* and *P* maize productivity. The larger the absolute values of these coefficients, the higher the influences of corresponding parameters on maize grain yield. The symbols “+” and “-” of each coefficient in equation (1) represent the positive and negative effects of *I*, *N* and *P* on maize grain yield respectively.

**Equation (2)~(4) and Figure 2 and 3: Individual effect**

Equations (2)~(4) are second-order parabolic curves and have apexes, which are also the maximums of maize grain yield as the rates of *I*, *N* and *P* increased (Figure 2).

The first-order differential analysis of Equations (2)~(4), also called incremental analysis, offers the cost-to-benefit ratio (i.e., marginal yield). The larger the marginal yield of *I*, *N* and *P*, the higher their respective influence on maize grain yield. The positive value of marginal yield indicates that maize grain yield increases with the increasing rate of *I*, *N* and *P*, while the negative value of marginal yield indicates that maize grain yield decreases with the increasing rate of *I*, *N* and *P*. When marginal yield is 0, yield function reaches its maximum value (Figure 3).

**Optimum schemes: The maximum of equations (1) and (5)**

Equation (1) can reach its maximum when$\frac{\partial Y}{\partial X_{1}}=\frac{\partial Y}{\partial X_{2}}=\frac{\partial Y}{\partial X_{3}}=0$.

By solving the partial differential analysis of equations (1), maximize maize grain yield can be obtained.

Similarly, the best economic profit was also solved using the method of first-order partial derivatives of equation (5).
